# The monofunctional CO dehydrogenase CooS is essential for growth of *Thermoanaerobacter kivui* on carbon monoxide

**DOI:** 10.1007/s00792-021-01251-y

**Published:** 2021-12-17

**Authors:** Surbhi Jain, Alexander Katsyv, Mirko Basen, Volker Müller

**Affiliations:** 1 Department of Molecular Microbiology and Bioenergetics, Institute of Molecular Biosciences, Johann Wolfgang Goethe University, Max-von-Laue-Str. 9, 60438 Frankfurt, Germany; 2 Microbiology, Institute of Biological Sciences, University of Rostock, 18059 Rostock, Germany

**Keywords:** Biofuels, Thermophilic acetogenic bacteria, Wood–Ljungdahl pathway, Synthesis gas

## Abstract

*Thermoanaerobacter kivui* is a thermophilic acetogen that can grow on carbon monoxide as sole carbon and energy source. To identify the gene(s) involved in CO oxidation, the genome sequence was analyzed. Two genes potentially encoding CO dehydrogenases were identified. One, *cooS*, potentially encodes a monofunctional CO dehydrogenase, whereas another, *acsA*, potentially encodes the CODH component of the CODH/ACS complex. Both genes were cloned, a His-tag encoding sequence was added, and the proteins were produced from a plasmid in *T. kivui*. His-AcsA copurified by affinity chromatography with AcsB, the acetyl-CoA synthase of the CO dehydrogenase/acetyl CoA synthase complex. His-CooS copurified with CooF_1_, a small iron-sulfur center containing protein likely involved in electron transport. Both protein complexes had CO:ferredoxin oxidoreductase as well as CO:methyl viologen oxidoreductase activity, but the activity of CooSF_1_ was 15-times and 231-times lower, respectively. To underline the importance of CooS, the gene was deleted in the CO-adapted strain. Interestingly, the ∆*cooS* deletion mutant did not grow on CO anymore. These experiments clearly demonstrated that CooS is essential for growth of *T. kivui* on CO. This is in line with the hypothesis that CooS is the CO-oxidizing enzyme in cells growing on CO.

**Supplementary Information:**

The online version contains supplementary material available at 10.1007/s00792-021-01251-y.

## Introduction

**Fig. 1:**
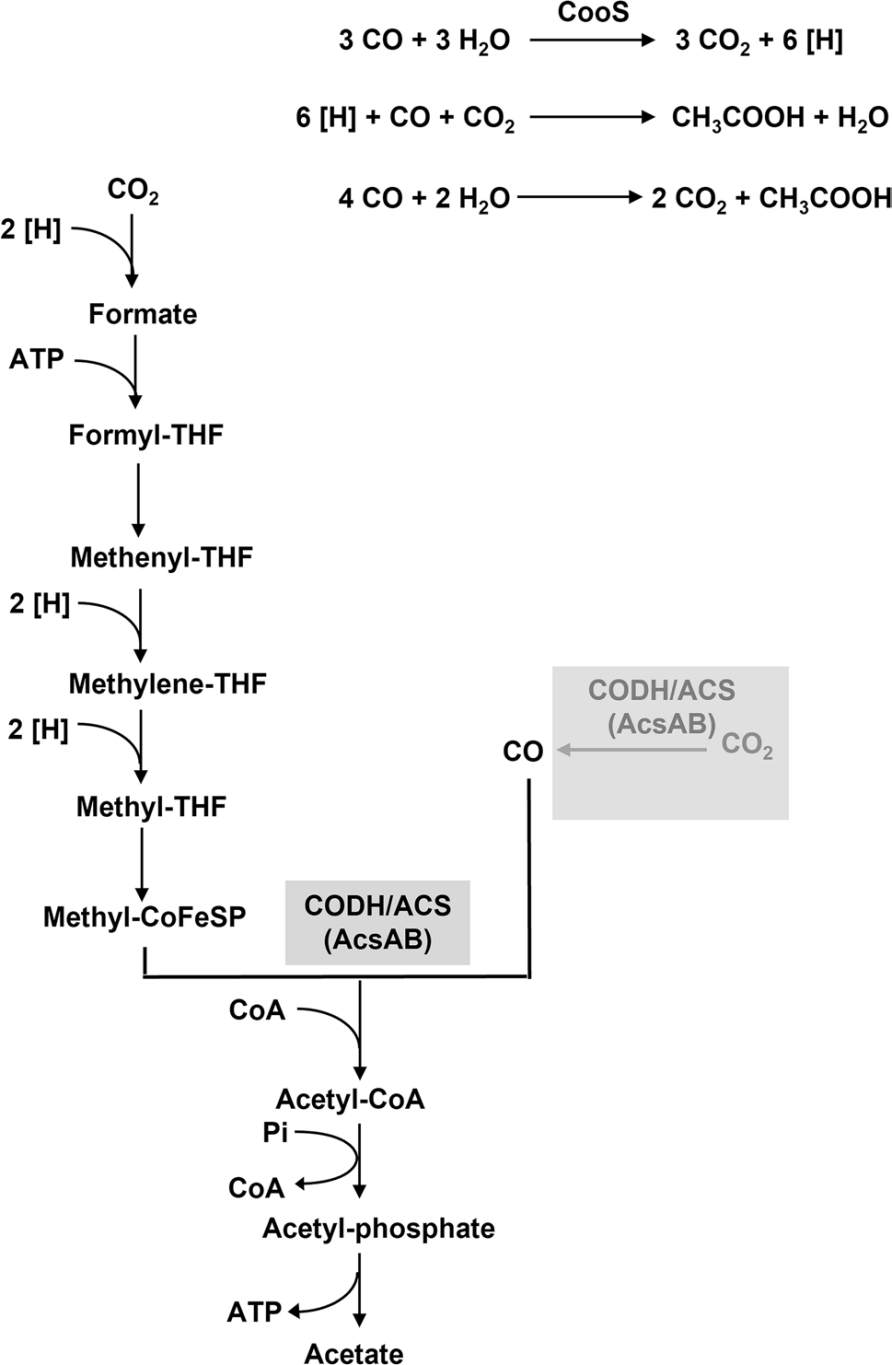
A simplified pathway for acetogenesis from CO. The monofunctional CO dehydrogenase, CooS is hypothesized to oxidize CO. The physiological function of the bifunctional CODH/ACS (AcsAB) is to reduce CO_2_ to CO which is then condensed with a methyl group and CoA to give acetyl-CoA. During growth on H_2_ + CO_2_ or organic substrates, CO is generated from CO_2_ by AcsAB (grey)

Carbon monoxide is an abundant atmospheric trace gas originating from biotic and abiotic sources (Stegenta-Dabrowska et al. 2019). The redox couple CO/CO_2_ has a rather negative redox potential (*E*_0_′[CO/CO_2_] = − 520 mV) (Thauer et al. 1977) and, thus, CO is an excellent electron donor for biological processes. Therefore, despite its high toxicity to many life forms, specialized bacteria and archaea are known to use carbon monoxide as electron and carbon source for growth (Henstra et al. 2007a; Sokolova et al. 2009; Robb and Techtmann 2018). These carboxydotrophs can be either aerobic or anaerobic but they share a common key enzyme, the CO dehydrogenase (CODH) (Ragsdale 2000; Dobbek et al. 2001). The enzyme is fundamentally different in aerobes and anaerobes (Doukov et al. 2002; Robb and Techtmann 2018). Aerobic bacteria have a molybdopterin as catalytic site for CO oxidation and channel the electrons into the quinone pool of a CO-insensitive electron transport chain (Wilcoxen et al. 2011; Meyer and Schlegel 1983). ATP is synthesized by electron transport phosphorylation and carbon is fixed by the Calvin cycle, allowing for a chemolithoautotrophic lifestyle (Meyer and Schlegel 1983). Anaerobic metabolism of CO is also chemolithoautotrophic, but the metabolism is different (Henstra et al. 2007b). The anaerobic CODH’s have a nickel atom as catalytic site and the enzymes involved can be either monofunctional (CooS) or bifunctional (AcsA) (Ragsdale 2000; Dobbek et al. 2001; Darnault et al. 2003). Monofunctional CODH’s are typically involved in CO oxidation, the direct electron acceptor is an iron sulfur center present in another protein (CooF), often encoded with *cooS* in an operon (Ensign and Ludden 1991; Kerby et al. 1992; Singer et al. 2006). The final electron acceptor may be a proton as in hydrogenotrophic carboxydotrophs (Henstra et al. 2007b), CO_2_ as in methanogenic archaea (Daniels et al. 1977; Rother and Metcalf 2004) or acetogenic bacteria (Diekert and Thauer 1978; Savage et al. 1987; Daniel et al. 1990; Diender et al. 2015) or sulfate as in sulfate reducing bacteria (Parshina et al. 2005) and archaea (Henstra et al. 2007a). Reduction of these electron acceptors lead to chemiosmotic energy conservation and the ATP gained is used to fix CO_2_, mainly by the Wood-Ljungdahl pathway (WLP) (Ragsdale and Wood 1985; Drake et al. 2008; Schuchmann and Müller 2014). In this two-branched, convergent pathway two molecules of CO_2_ are reduced to acetyl-CoA. The key enzyme of the WLP is the CODH/ACS that has a bifunctional CODH whose cellular function is to catalyze the reversal of the aforementioned reaction, the reduction of CO_2_ to CO, the precursor of the carboxyl group in acetyl-CoA (Diekert and Thauer 1978; Savage et al. 1987; Daniel et al. 1990; Diender et al. 2015; Schuchmann and Müller 2014) (Fig. 1). However, in vitro, the bifunctional CO dehydrogenase also catalyzes oxidation of CO (Carlson and Papoutsakis 2017).

Acetogenic bacteria are of outstanding biotechnological interest for a sustainable bioeconomy (Schiel-Bengelsdorf and Dürre 2012; Bertsch and Müller 2015; Müller 2019; Katsyv and Müller 2020), since they can convert carbon dioxide and molecular hydrogen or carbon monoxide or a combination thereof (synthesis gas; syngas) to valued-added chemicals (Demler and Weuster-Botz 2010; Dürre 2011; Köpke et al. 2011; Liew et al. 2016b; Wilkins and Atiyeh 2011). Some mesophilic acetogens are already used as biocatalysts for ethanol production from syngas (Najafpour and Younesi 2006; Maddipati et al. 2011; Bertsch and Müller 2015). In this process, carbon monoxide is oxidized alongside with molecular hydrogen. Recently, we have established that the thermophilic acetogen *Thermoanaerobacter kivui*, that had been described not to grow on CO (Daniel et al. 1990), can be adapted to grow on CO by serial transfers to media with increasing CO concentrations (Weghoff and Müller 2016). This bacterium also grows on CO or syngas in mineral media making it an ideal biocatalyst for CO or syngas-derived valued-added chemicals (Müller 2019). Recently, a procedure was established enabling *T. kivui* to convert carbon monoxide to formate, a very promising approach for CO conversion (Schwarz et al. 2020). The molecular basis of adaptation to CO as well as the enzyme involved in using CO as carbon and electron source remained elusive. Here, we have used a genetic approach to identify the CO dehydrogenase involved in growing on CO.

## Materials and methods

### Organisms and cultivation

*T. kivui* strains listed in Table 1 were routinely cultivated under anoxic conditions at 66 °C in complex or defined media described before (Weghoff and Müller 2016; Basen et al. 2018). All the growth experiments were performed in 120 ml serum bottles (Glasgerätebau Ochs GmbH, Bovenden-Lenglern, Germany) containing 20 or 50 ml media for growth on gases or sugars. For growth on CO, the gas phase was 100% CO (2 × 10^5^ Pa), for growth on 25 mM glucose, the atmosphere was N_2_/CO_2_ (80/20 [v/v], 1 × 10^5^ Pa). Growth was monitored by measuring the OD at 600 nm. Plating and cultivation method on solid media was the same as described in (Basen et al. 2018).

**Table 1:** Strains used in this study

Strain	Deleted gene	Parent strain	Reference of parent strain
Wild type *T. kivui* (CO)*	–	DSM 2030	Leigh et al. (1981)
TKV_SJ001	*cooS* (TKV_08080)	*T. kivui* Δ*pyrE* (TKV002)	Basen et al. (2018)
*T. kivui* Δ*pyrE* (CO)	*pyrE* (TKV_c14380)	wild type *T. kivui* (CO)	Weghoff and Müller (2016)
*T. kivui* Δ*cooS* (CO)	*cooS* (TKV_08080)	*T. kivui* Δ*pyrE* (CO)	This study

^*^(CO) denotes that the strain was adapted to growth on CO

### Production and purification of His-CooS and His-AcsA

Plasmid *pMU131_His-cooS* and *pMU131_His-acsA* were used for the expression of *cooS* (TKV_c08080) and *acsA* (TKV_c20100) in *T. kivui*. The plasmids are based on plasmid *pMU131* (Shaw et al. 2010) which is replicating in *T. kivui* and confers resistance to kanamycin (Basen et al. 2018). The inserts *His-cooS* (size: 1954 bp) and *His-acsA* (size: 1927 bp) were amplified using the primers His_CooS_for (3) (5′- CAAGGAGGAGGATTGACTGTATGCACCATCATCATCACCATCATCATCATCATAGTGATAATTACATTTATTCTGCTG) and His_CooS_rev (4) (5′-TCC TGGATAAATTTAAAAAATTATATATTGAGTAGTTTGCGC) or His_AcsA_for (5) (5′- CAAGGAGGAGGATTGACTGTATGCACCATCATCATCACCATCATCATCATCATGAAGAGAAGAGAACTATTGACATTG) and His_AcsA_rev (6) (5′-TCCTGGATAAATTTA AAAAATTAAACACCCAAAGCCTTTC). The backbone *pMU131* (size: 7192 bp) was amplified using the primers pMU131_for (1) (5′-TTTTTTAAATTTATCCAGGATAAAAGAGAAGACTC) and pMU131_rev (2) (5′-ACAGTCAATCCTCCTCCTTG), followed by the fusion of the PCR products *via* Gibson Assembly (Gibson Assembly Mastermix, NEB, Frankfurt/Main, Germany). *T. kivui* (DSM 2030) was transformed with the plasmids *pMU131_His-cooS* or *pMU131_His-acsA* as described (Basen et al. 2018). Cells were plated on agar medium containing 28 mM glucose as carbon source and 200 µg/ml kanamycin as selection marker. To verify the transformation, colonies were picked and the transformed plasmids were checked by using primer pairs seq1_for (7) (5′-TCTAACACAATTATATCATAAGGATTG ATA)/seq2_rev (8) (5′-AGTATTGTCAATATATTCAAGGCAA) binding on the *pMU131* backbone and amplifying the complete *His-cooS* or *His-acsA* locus (Fig. S1).

For the purification of the His-tagged CooS or AcsA, *T. kivui pMU131_His-cooS* or *pMU131_His-acsA* cells were grown with 28 mM glucose as carbon and energy source and 200 µg/ml kanamycin. All purification steps were performed under strictly anoxic conditions at room temperature in an anoxic chamber (Coy Laboratory Products, Grass Lake, Michigan, USA) filled with 95–98% N_2_ and 2–5% H_2_. *T. kivui* cells were harvested and washed twice in buffer A (50 mM Tris/HCl, 150 mM NaCl, 20 mM MgSO_4_, 10 mM imidazole, 0.5 mM DTE, 4 µM resazurin, 20% [v/v] glycerol, pH 7.5). The cells were resuspended in 50 ml buffer A including 0.5 mM PMSF and 0.1 mg/ml DNAseI and passed one time through a French pressure cell (110 MPa). Cell debris was removed by centrifugation at 24,000×*g* for 20 min. Protein purification of the His-tagged proteins was carried out on a nickel nitrilotriacetic acid (Ni^2+^-NTA) resin (Qiagen, Hilden, Germany) using a gravity flow column under anoxic conditions as described previously (Katsyv et al. 2021). Fractions containing His-CooS or His-AcsA were collected, pooled, concentrated, using 50-kDa VIVASPIN tubes, and separated on a Superdex 200 10/300 GL increase prepacked column (GE Healthcare Life Sciences, Little Chalfont, UK). The sample was loaded on a Superdex 200 column equilibrated with buffer B (50 mM Tris/HCl, 150 mM NaCl, 20 mM MgSO_4_, 2 mM DTE, 4 µM resazurin, 20% [v/v] glycerol, pH 7.5) and eluted at a flow rate of 0.5 ml/min. CooS and AcsA activity eluted in a single peak with a maximum at 9.7 and 12.6 ml elution volume, respectively. Fractions containing His-CooS and His-AcsA were pooled and stored at 4 °C.

### Preparation of cell free extract

Cells were cultivated in 1 L flasks (Müller-Krempel, Bülach, Switzerland) filled with 200 ml or 500 ml of complex media for growth on 100% CO (2 × 10^5^ Pa) or 25 mM glucose, respectively. Mid exponential phase cells were harvested by centrifugation at 11,500 × g for 10 min at 4 °C and washed twice in buffer C (50 mM Tris/HCl, 20 mM MgSO_4_, 20% glycerol, 2 mM DTE, 4 μM resazurin, pH 7). After the washing steps, cells were resuspended in 20 ml buffer C with few crystals of DNAseI and 0.5 mM PMSF and disrupted by a passage through a French pressure cell (110 MPa). Cell debris was separated by centrifugation at 14,300 × g for 20 min and the supernatant was collected. The supernatant contained the cell free extract.

### Measurement of CODH activity

Enzyme assays were routinely performed at 66 °C in 1.8 ml anoxic cuvettes (Glasgerätebau Ochs, Bovenden-Lenglern, Germany) sealed by rubber stoppers in a 100% CO atmosphere (2 × 10^5^ Pa) with buffer D (50 mM Tris/HCl, 10 mM NaCl, 2 mM DTE, 4 μM resazurin, pH 7.5 or pH 7) or buffer E (100 mM HEPES/NaOH, 2 mM DTE, 2 μM resazurin, pH 7) at an overall liquid volume of 1 ml. His-CooSF_1_ or His-AcsAB activity was measured with methyl viologen (MV) or ferredoxin (Fd) as electron acceptor at 604 nm (*ε* = 13.9 mM^−1^ cm^−1^) or 430 nm (*ε* = 13.1 mM^−1^ cm^−1^), respectively. Fd was purified from *Clostridium pasteurianum* as described previously (Schönheit et al. 1978). CODH activity in cell free extracts was measured in buffer E using MV as an artificial electron acceptor. The assay was supplemented with crude extract or enriched His-CooSF_1_ or His-AcsAB preparation and the reaction was started with 10 mM MV or 30 μM Fd. For *K*_m_ determination, the CO and Fd concentrations ranged between 0–300 µM and 0–200 µM, respectively. For the determination of the pH and temperature profile, the assay mixture including the protein was preincubated for 10 min at the pH or temperature indicated. The buffer (F) used to determine the pH optima was 50 mM MES, 50 mM CHES, 50 mM CAPS, 50 mM Bis–Tris, 50 mM Tris, 10 mM NaCl, 4 mM DTE, 4 μM resazurin.

### Analytical methods

The concentration of proteins was measured according to Bradford (1976). The protein concentration in whole cells was measured according to Schmidt (Schmidt et al. 1963). The iron content of the purified enzymes was determined by colorimetric methods (Fish 1988). Proteins were separated in 12% polyacrylamide gels and stained with Coomassie brilliant blue G250. The molecular mass of the purified His-CooSF_1_ or His-AcsAB was determined using a calibrated Superdex 200 column, buffer B and defined size standards (ovalbumin: 43 kDa; albumin: 158 kDa; catalase: 232 kDa; ferritin: 440 kDa).

### Generation of *T. kivui* Δ*cooS* strain

For the deletion of *cooS* (Tkv_c08080), plasmid *pSJ006* (Fig. S2A) was constructed in *E. coli DH5α*. The plasmid was generated by inserting 1000 bp upstream flanking region (UFR) and downstream flanking region (DFR) of *cooS* into the backbone plasmid *pMBTkv0012* (Jain et al. 2020). Primers used were ∆*cooS*_UFR_FP (5′- ACCCGGGGATCCGCAGGAAGATTGGAAGTCAT) & ∆*cooS*_UFR_RP (5′- CCCATATTTTTCAATTATTATCACAACTCCTTTT) for UFR and ∆*cooS*_DFR_FP (5′- GGAGTTGTGATAATAATTGAAAAATATGGGAGGAA) & ∆*cooS*_DFR_RP (5′- GCAGGTCGACTCTAGACTGGTCGGGGCAACAGGAT) for DFR amplification, followed by ligation into *pMBTkv0012* using oligonucleotides ∆*cooS*_BB_FP (5′- GCCCCGACCAGTCTAGAGTCGACCTGCAGGCATG) & ∆*cooS*_BB_RP (5′- CAATCTTCCTGCGGATCCCCGGGTACCGAGCTCG).

*T. kivui* Δ*pyrE* (Basen et al. 2018) was transformed with the plasmid *pSJ006*. Since the plasmid contains *pyrE* gene as a selection marker, the first round of selection was performed on agar plates in defined media without uracil using 25 mM glucose as a substrate to select for transformants with the plasmid integration. Further, these transformants were subjected to second round of selection in media supplemented with 25 mM glucose, 50 µM uracil and 5 mM 5-fluoroorotate (5-FOA) to select the isolates with the loss of plasmid. The deletion of *cooS* was confirmed by PCR of isolated genomic DNA using forward primer (5′- GGGCTTTATAAAGCGAAATGGG) and reverse primer (5′- GCCTGTTGATAAGTCATAAAACCTGC) binding outside the *cooS* gene or using forward primer (5′- GCGTGATCCAAAATGTGGTTTCGG) and reverse primer (5′- CAAGCCATTGTGGTGCAGAAGC) binding inside the *cooS* gene in the genome.

For the requirement of ∆*pyrE* in the CO adapted strain, plasmid *pMBTkv002b* (Basen et al. 2018) was used. The CO adapted wild type (Weghoff and Müller 2016) was transformed with *pMBTkv002b* and subjected to 5-FOA selection with 50 µM uracil. The deletion of *pyrE* was verified by primers MB_IG_0005 and MB_IG_0006 (Basen et al. 2018). To delete the *cooS* in the generated CO adapted ∆*pyrE*, plasmid *pSJ006* was used as described above. The deletion of *cooS* was confirmed by amplifying the flanking regions followed by DNA sequencing analysis (Sanger et al. 1977).

To integrate *cooS* gene back into the *T. kivui* ∆*coos* genome, plasmid *pSJ008* was prepared (Fig. S2B). *pJM006* originated from *pMBTkv007* (Basen et al. 2018), was used as the backbone plasmid, which has *pyrE* gene under control of the promoter gyrase from *Thermoanaerobacter* sp. strain X514, and directly adjacent to the 3′-end, gene Teth514_0627 from *Thermoanaerobacter* sp. strain X514 under control of the promoter of the S-layer protein from *T. kivui*. *pJM006* except for *adhE* was amplified by PCR using primers (5′- CTACTCAATATATAAAATTTAATTTAAAAATTTCACAGCAAGCAG) and (5′- TGTAATTATCACTCATACAGTCAATCCTCCTCCTTGTATTTG). The *cooS* gene was amplified by using forward primer (5′- GGAGGATTGACTGTATGAGTGATAATTACATTTATTCTGCTG) and reverse primer (5′- GTGAAATTTTTAAATTAAATTTTATATATTGAGTAGTTTGCGCC). The PCR products were then fused to generate the plasmid *pSJ008* using Gibson assembly (Gibson Assembly Mastermix, NEB, Frankfurt/Main, Germany). *T. kivui* ∆*cooS* mutant was transformed with plasmid *pSJ008*. Selection for the transformants was performed by using defined media without uracil in the presence of 25 mM glucose.

### Preparation of resting cells

Preparation of resting cells were performed under anoxic condition. Cells of *T. kivui* wild type and ∆*cooS* were grown on glucose or on glucose + 100% CO in the headspace, in 500 ml of complex media to mid exponential phase and were harvested by centrifugation (AvantiTMJ-25 and JA-10 Fixed-Angle Rotor; Beckman Coulter, Brea, CA, United States) at 11,500 × g, 4 °C for 10 min. The supernatant was discarded and the cells were washed three times with imidazole buffer (50 mM imidazole, 20 mM MgSO_4_, 20 mM KCl, 20 mM NaCl, 4 mM DTE, 4 μM resazurin, pH 7). After centrifugation, cells were resuspended in 5 ml of same buffer and kept in 16 ml gas tight Hungate tubes. The headspace of the Hungate tubes were changed to 100% N_2_. The protein concentration was measured according to (Schmidt et al. 1963).

### Experiment with resting cells

For the experiment, 120 ml serum bottles (Glasgerätebau Ochs GmbH, Bovenden-Lenglern, Germany) under a N_2_/CO_2_ (80/20 [v/v], 1 × 10^5^ Pa) atmosphere were filled with imidazole buffer (50 mM imidazole, 20 mM MgSO_4_, 20 mM KCl, 20 mM NaCl, 4 mM DTE, 4 μM resazurin, pH 7) in the presence of 50 mM of KHCO_3_. Cells were added to a protein concentration of 1 mg/ml, the final volume of the suspension was 10 ml. After the serum bottles were incubated at 66 °C for 10 min in pre-warmed water bath, the experiment was started by addition of H_2_ + CO_2_ (80/20 [v/v], 1 × 10^5^ Pa).

### Product analysis

Acetate production was measured by gas chromatography as described previously (Weghoff and Müller 2016).

## Results

### Identification and organization of genes involved in CO metabolism

**Fig. 2:**
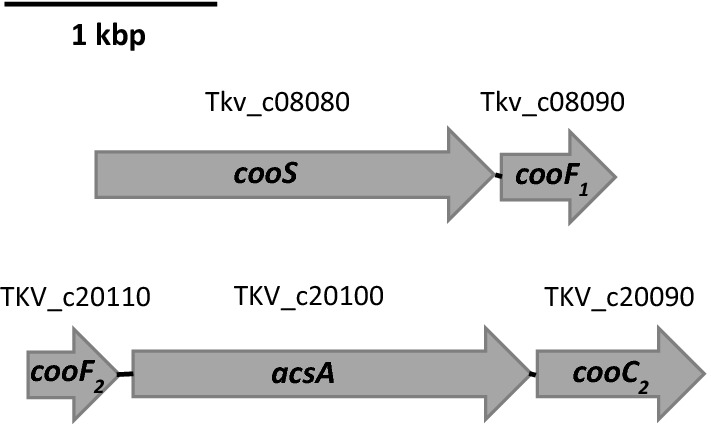
Genetic organization of CO dehydrogenase genes in *T. kivui*. Genes coding for potential monofunctional CODH in *T. kivui*, *cooS*, organized with *cooF*_*1*_ (upper panel), and a second CODH, *acsA* adjacent to *cooF*_*2*_ and *cooC*_*2*_ (lower panel)

To identify genes that are involved in CO metabolism, the genome sequence of *T. kivui* was inspected. The genome harbors one gene encoding a potential monofunctional CODH, *cooS* (Tkv_c08080) (Hess et al. 2014). The downstream region is flanked by the gene *cooF*_*1*_ (Tkv_c08090), potentially involved in CO metabolism for transferring electrons to a membrane-bound hydrogenase (Fox et al. 1996; Schoelmerich and Müller 2020) (Fig. 2). CooF_1_ has a predicted molecular mass of 19 kDa and contains three 4Fe-4S cluster. Upstream of *cooS* is a hypothetical gene with unknown function. In silico analysis using the BlastP algorithm revealed 54, 32, 34, 53 and 52% identity of CooS of *T. kivui* to CooS of *Caldicellulosiruptor hydrothermalis, Rhodospirillum rubrum, Desulfovibrio vulgaris, Clostridium carboxidivorans* and *Acetobacterium woodii*, respectively. CooS has a predicted molecular mass of 68 kDa and contains a 4Fe-4S cluster and a Ni-4Fe-4S center where carbon monoxide oxidation occurs (Ragsdale and Kumar 1996). Additionally, the genome of *T. kivui* contains another putative CODH gene annotated as *acsA* (TKV_c20100) that together with *acsB* (TKV_c19820), encoding the acetyl-CoA synthase, forms the CODH/ACS complex. The gene *acsA* is flanked by a second copy of *cooF* (*cooF*_2_) (TKV_c20110), and a gene encoding potentially for nickel insertion, *cooC*_*2*_ (TKV_c20090) (Fig. 2), all transcribed in the same direction. AcsA is predicted to have a molecular mass of 67 kDa and has as well a 4Fe-4S cluster and Ni-4Fe-4S center, sharing 61 and 60% identity to *Methanosarcina mazei* and *Thermoanaerobacter* sp. YS13, respectively.

### Purification and characterization of His-CooS and His-AcsA

**Fig. 3:**
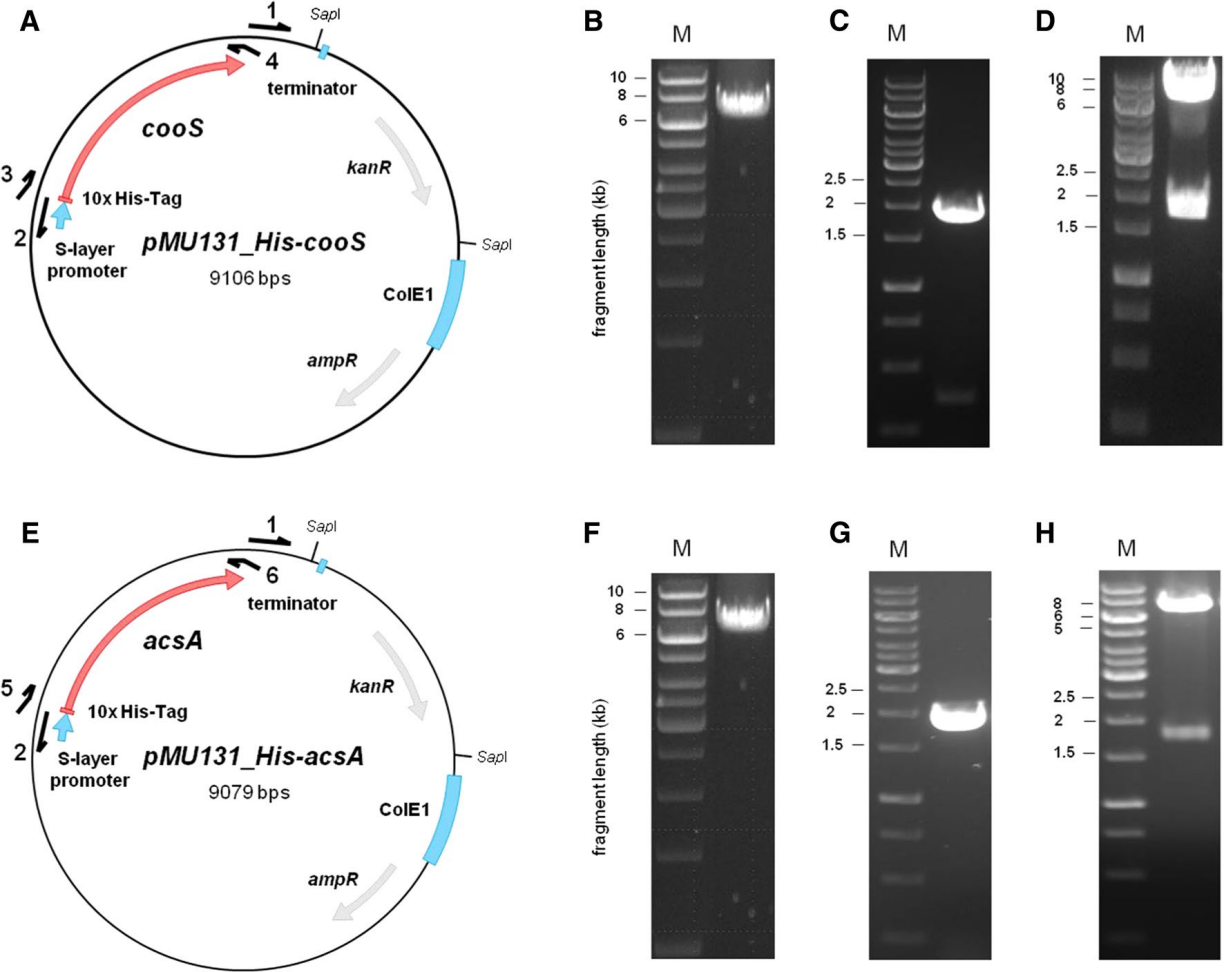
Cloning of *pMU131_ His_cooS* and *pMU131_His_acsA*. For the production of His-CooS and His-AcsA in *T. kivui* the constructs *pMU131_ His-cooS* and *pMU131_His-acsA* were cloned (**A**, **E**). Therefore, *pMU131* backbone, including a S-layer-promoter, was amplified using corresponding primers *via* PCR (**B**, **F**). *His*-*cooS* (**C**) and *His-acsA* (**G**) were amplified from genomic DNA of *T. kivui via* PCR, using corresponding primers, containing an additional DNA sequence coding for a 10 × His-tag. Amplified *His-cooS* or *His-acsA* and *pMU131* backbone were fused *via* Gibson Assembly and transformed in *E. coli* HB101. Afterwards, plasmids were isolated and digested with *SacI* (**D**, **H**). The resulting sizes for *pMU131_ His-cooS* were 7253 bp and 1826 bp and for *pMU131_His-acsA* 7280 bp and 1826 bp. M, Gene Ruler 1 kb DNA ladder

**Fig. 4:**
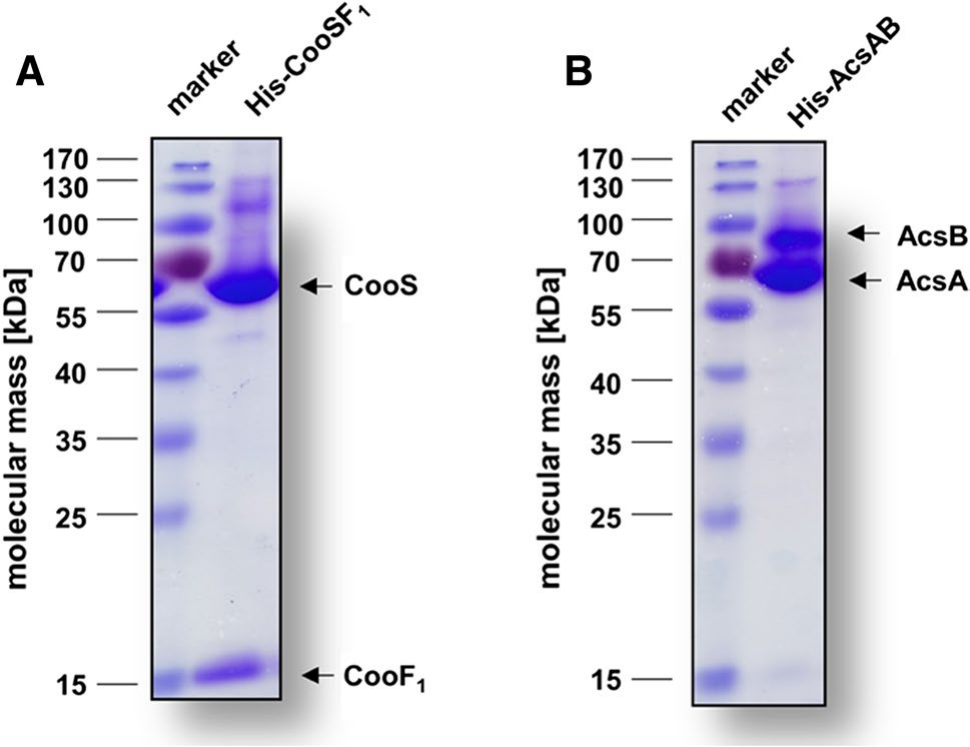
SDS-PAGE of the purified His-CooSF_1_ and His-AcsAB complex. His-CooSF_1_ (**A**) and His-AcsAB (**B**) of *T. kivui*, purified *via* affinity chromatography and separated on a gelfiltration column, were analyzed in a denaturating SDS-PAGE (12%). The proteins were stained with Coomassie Brilliant Blue G250. 10 µg of protein was applied to each lane. M, prestained page ruler

In order to establish that CooS and AcsA are indeed CO dehydrogenases, we took advantage of a plasmid-based production system in *T. kivui* (Katsyv et al. 2021). Therefore, we cloned *cooS* (TKV_c08080) or *acsA* (TKV_c20100) together with a DNA sequence coding for a 10 × histidine-tag into *pMU131*, which replicates in *T. kivui* (Fig. 3). Naturally competent cells of *T. kivui* (DSM 2030) were transformed with the plasmid and the encoded proteins containing a genetically engineered His-tag were purified. Therefore, crude extract of the genetically modified *T. kivui* strains was prepared as described in Material and Methods. The His-tagged CooS and AcsA were purified from the crude extract to apparent homogeneity by Ni^2+^-NTA-sepharose followed by a size exclusion chromatography on Superdex 200. Analyses of the purified His-CooS separated on a 12% SDS–polyacrylamide gel revealed two proteins with apparent molecular masses of ≈ 65 and ≈ 17 kDa (Fig. 4A). These molecular masses correspond well with the expected sizes for CooS (TKV_c08080, 68 kDa) and the downstream encoded CooF_1_ (TKV_c08090, 19 kDa) of *T. kivui*. Analytical size exclusion chromatography revealed a molecular mass of 537 kDa for the purified complex, which is consistent with His-CooSF_1_ being a hexamer. We measured 22.9 ± 0.6 mol of iron/mol of protein using the colorimetric assay to detect complexed iron (Fish 1988), which matches the prediction that His-CooSF_1_ contains four 4Fe-4S cluster and one Ni-4Fe-4S. When analyzing the enriched His-AcsA protein on a 12% SDS–polyacrylamide gel, two proteins with apparent molecular masses of ≈ 65 and ≈ 80 kDa were visible (Fig. 4B). These molecular masses correspond well with the expected sizes for AcsA (TKV_c20100, 67 kDa) and AcsB (TKV_c19820, 76 kDa) of *T. kivui*. Apparently, AcsA and AcsB form a stable complex that can be purified *via* affinity chromatography of AcsA. Stable complex formation of the CODH subunit (AcsA) with the acetyl-CoA synthase (AcsB) was shown before (Ragsdale et al. 1983; Ragsdale and Kumar 1996; Seravalli et al. 1997; Doukov et al. 2008). Analytical size exclusion chromatography revealed a molecular mass of 130.5 kDa for the purified complex, which is consistent with His-AcsAB being a monomer. We measured 9.0 ± 0.3 mol of iron/mol of protein, which matches the prediction that His-AcsAB contains one 4Fe-4S cluster and one Ni-4Fe-4S.

Both complexes exhibited CO:MV oxidoreductase as well as CO:Fd oxidoreductase activity (Table 2). The specific CO:MV oxidoreductase activity of His-AcsAB was 213.9 ± 7.6 U/mg, that of His-CooSF_1_ was 15-times lower with 13.9 ± 4.1 U/mg (Table 2). The bifunctional as well as the monofunctional CODH reduced Fd (isolated from *C. pasteurianum*) as electron acceptor with CO as electron donor. His-CooSF_1_ catalyzed CO:Fd oxidoreductase activity significantly lower with 0.5 ± 0.03 U/mg compared to His-AcsAB with 111.5 ± 15.4 U/mg (Table 2). Since this is the first purification of a monofunctional and bifunctional CODH from one organism, it is unknown whether the huge difference in activity is a unique feature of the enzymes for *T. kivui*.

**Table 2:** CODH activities determined with purified His-AcsAB and His-CooSF1

	His-AcsAB [U/mg]	His-CooSF_1_ [U/mg]
CO:MV oxidoreductase activity	213.9 ± 7.6	13.9 ± 4.1
CO:Fd oxidoreductase activity	111.5 ± 15.4	0.5 ± 0.03

Enzyme assays were routinely performed at 66 °C in 1.8 ml anoxic cuvettes sealed by rubber stoppers in a 100% CO atmosphere (2 × 10^5^ Pa) with buffer D (50 mM Tris/HCl, 10 mM NaCl, 2 mM DTE, 4 μM resazurin, pH 7.5 or pH 7). The assay was supplemented with enriched His-CooSF_1_ or His-AcsAB preparation and the reaction was started by addition of 10 mM MV or 30 μM Fd. The measurements were carried out in biological triplicates (*n* = 3)

We assessed key biochemical properties of the purified His-AcsAB and His-CooSF_1_, including temperature and pH stability, substrate affinities and cofactor dependence. To ensure an ideal reflection of the physiological conditions, we exclusively used the CO:Fd oxidoreductase assay. His-AcsAB was active at temperatures ranging from 22 to 85 °C with a maximal activity of 100.6 ± 7.8 U/mg at the optimal growth temperature of *T. kivui* (66 °C) (Fig. S3A). His-AcsAB activity decreased by 96% at 22 °C and by 65% at 40 °C (Fig. S3A). The pH range was relatively narrow with only 5% activity at pH 6 and 10 and an optimal activity of 48.5 ± 4.1 U/mg at pH 7 and 8 (Fig. S3B).

His-CooSF_1_ was active at temperatures ranging from 22 to 85 °C with a maximal activity of 0.5 ± 0.03 U/mg at the optimal growth temperature of *T. kivui* (66 °C) (Fig. S4A). His-AcsAB activity was decreased by 83% at 22 °C and by 62% at 40 °C (Fig. S4A). Compared to His-AcsAB, His-CooSF_1_ was almost fully active at temperatures higher than 66 °C (Fig. S4A). The pH range was relatively narrow with zero activity at pH 5 and 10. At pH 6 CO:Fd oxidoreductase activity was 0.2 ± 0.01 U/mg and reached an optimum of 0.3 ± 0.03 U/mg at pH 7 (Fig. S4B). All further analyses were subsequently carried out at pH 7.5 for His-AcsAB and pH 7 for His-CooSF_1_ and 66 °C, to ensure optimal enzyme activity.

Next, we assessed the K_m_ values for all reaction partners of AcsAB and CooSF_1_. The dependence of the CO:Fd oxidoreductase reaction on CO and Fd was hyperbolic with saturation at ~ 30 µM CO or 30 µM Fd for His-AcsAB and His-CooFS_1_ (Fig. S3C, D and Fig. S4C, D). The Km values of His-AcsAB for CO and Fd were 10.9 ± 3.6 µM and 15.9 ± 4.6 µM, respectively (Fig. S3C, D). The Km values of His-CooSF_1_ for CO and Fd were 5.0 ± 1.5 µM and 20.9 ± 6.0 µM, respectively (Fig. S4C, D). Unsurprisingly, the absence of any reaction partner led to a complete loss of activity for both CODHs.

### Generation of *cooS *deletion in *T. kivui*

Since previous data revealed higher level of CooS compared to AcsA during growth on CO (Weghoff and Müller 2016), we aimed to delete *cooS* (Tkv_c08080) and study its involvement in growth on CO. Therefore, plasmid pSJ006 was generated containing approximately 1000 bp upstream and downstream of the *cooS* gene. The plasmid also contained the *pyrE* cassette as selectable marker that can be integrated into the *pyrE*-deficient uracil-auxotrophic strain TKV_MB002 (Basen et al. 2018) at one of the flanking regions. The subsequent disintegration was forced by the presence of 5-FOA since the plasmid contains a *pyrE* gene for production of a functional orotate phosphoribosyltransferase, leading to markerless deletion of *cooS*. Indeed, with glucose as carbon and energy source for growth of *T. kivui*, we were able to delete the *cooS* gene (data not shown). This strain is named as TKV_SJ001, to avoid confusion.

However, we could not adapt TKV_SJ001 strain to grow on CO, using the same procedure as described previously (Weghoff and Müller 2016). The failure to grow the mutant on CO could reflect the essentiality of CooS for CO metabolism or the inability to adapt the cells to the toxic gas. Therefore, we used a different approach and decided to delete *cooS* in a CO-adapted strain [∆*cooS* (CO)]. Thus, *pyrE* had to be deleted first; this was done essentially as described above, in the CO-adapted wild type *T. kivui*. In order to ease the mutant preparation on solid media, we made sure that the CO-adapted strain would start to grow on CO immediately when cultivated on glucose in between. Then, the *cooS* gene was deleted, essentially as described above using the same approach with cells grown on glucose. Again, we were able to delete the *cooS* gene, as exemplified by PCR with primers binding outside the *cooS* gene (Fig. S5A). Isolate 1 was additionally verified by PCR with the primers binding inside the *cooS* gene and as expected, an amplificate was not obtained whereas, with DNA from the parental strain ∆*pyrE* (CO) a DNA fragment of 1.5 kb was amplified (Fig S5B). All strains used in this work are listed in Table 1. To avoid confusion, from now on the ∆*cooS* strain in this work refers to the CO-adapted *T. kivui* ∆*pyrE* strain with a *cooS* deletion, if not otherwise specified.

### The *cooS* mutant does not grow on carbon monoxide

**Fig. 5:**
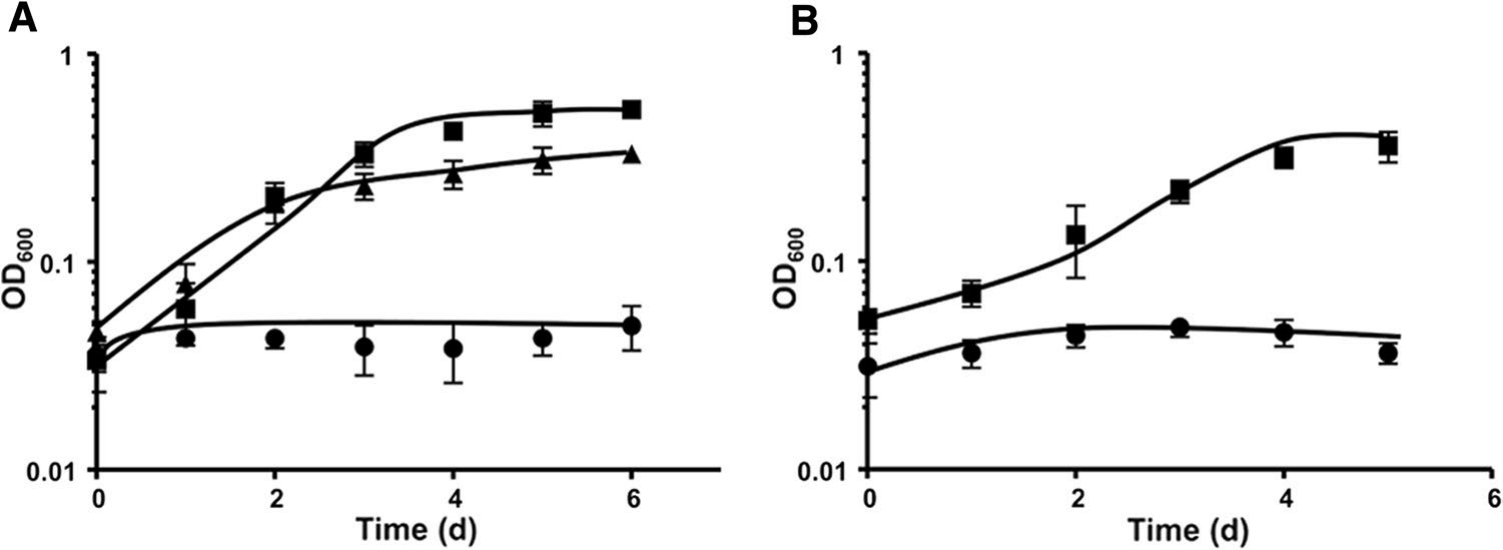
Growth of *T. kivui* Δ*pyrE* (CO) and Δ*cooS* on 100% CO. The cells were grown at 66 °C with 100% CO (2 × 10^5^ Pa) in 120 ml serum bottle containing 20 ml of complex media (**A**) or mineral media with uracil (**B**). These experiments were performed in biological triplicates. Squares, *T. kivui* Δ*pyrE* (CO); circles, *T. kivui* Δ*cooS*; triangles, *T. kivui* Δ*cooS* plus re-introduced *cooS* in a different genome location

To analyze the phenotype of the *cooS* mutant growth experiments were performed. The ∆*cooS* strain grew on 25 mM glucose, 25 mM mannitol, H_2_ + CO_2_ (2 × 10^5^ Pa) or 100 mM formate, similar to the parent strain ∆*pyrE* (CO). The ∆*pyrE* (CO) strain grew on 100% CO with a rate of 0.012 (h^−1^) to an OD of 0.54 ± 0.04 (*n* = 3) after 5 days in complex media. In contrast, the ∆*cooS* mutant did not grow in the time frame observed. When the *cooS* gene was presented *in trans* on plasmid pSJ008 cells did grow on CO with rates and final yields were comparable to the ∆*pyrE* (CO) (Fig. 5A).

However, after 7 days the ∆*cooS* mutant started to grow with a rate of 0.0081 (h^−1^) to a final OD of 0.28 ± 0.06 (*n* = 3). In contrast, this was not observed in mineral media, indicating that cells of the ∆*cooS* mutant did not grow on CO but on a component of the complex media. When the cells were transferred from glucose-mineral medium to CO-mineral medium, slow growth was observed after 15 days leading to a final OD of 0.2, but after a second transfer, growth was no longer observed. In contrast, when ∆*pyrE* (CO) was transferred, it grew to an OD of 0.35 ± 0.06 (*n* = 3) in 5 days in mineral media (Fig. 5B).

To determine the CO dehydrogenase activity in ∆*pyrE* (CO) and the ∆*cooS* mutant, both were grown on CO in complex media (the ∆*cooS* for 7 days). Cells were harvested in late exponential growth phase, cell free extract was prepared and the CODH activity was determined with CO as electron donor and MV as electron acceptor (Table 3). The CO:MV oxidoreductase activity was 178.6 ± 12.8 U/mg in ∆*pyrE* (CO) but only 8% (14.9 ± 2.5 U/mg) in the ∆*cooS* mutant, clearly demonstrating that the majority of CODH activity is catalyzed by CooS. Complementation of the mutant restored CODH activity by 41.6% to 76.9 ± 6.9 U/mg. When the CO dehydrogenase activity was measured in glucose-grown cells, similar activities were observed in the ∆*cooS* mutant (50.6 ± 6.1 U/mg) and ∆*pyrE* (CO) (53.8 ± 11.4 U/mg).

**Table 3:** CODH activities determined in cell free extract of ∆*pyrE* strain (CO), ∆*cooS* mutant and *cooS* complemented strain

Strain	Conditions	
	100% CO (U/mg)	Glucose (U/mg)
*T. kivui* ∆*pyrE* (CO)	178.6 ± 12.8	50.6 ± 6.1
*T. kivui* ∆*cooS*	14.9 ± 2.5	53.8 ± 11.4
*T. kivui* ∆*cooS* plus re-introduced *cooS*	76.9 ± 6.9	107 ± 2.3

Cells were grown on 100% CO or on 25 mM glucose and harvested in the in mid-exponential growth phase. Enzyme assays were routinely performed at 66 °C in 1.8 ml anoxic cuvettes sealed by rubber stoppers in a 100% CO atmosphere (2 × 10^5^ Pa) with buffer E (100 mM HEPES/NaOH, 2 mM DTE, 2 μM resazurin, pH 7). The assay was supplemented with the cell free extract and the reaction was started with 10 mM MV. The measurements were carried out in biological triplicates (*n* = 3)

### Cell suspension experiments

**Fig. 6:**
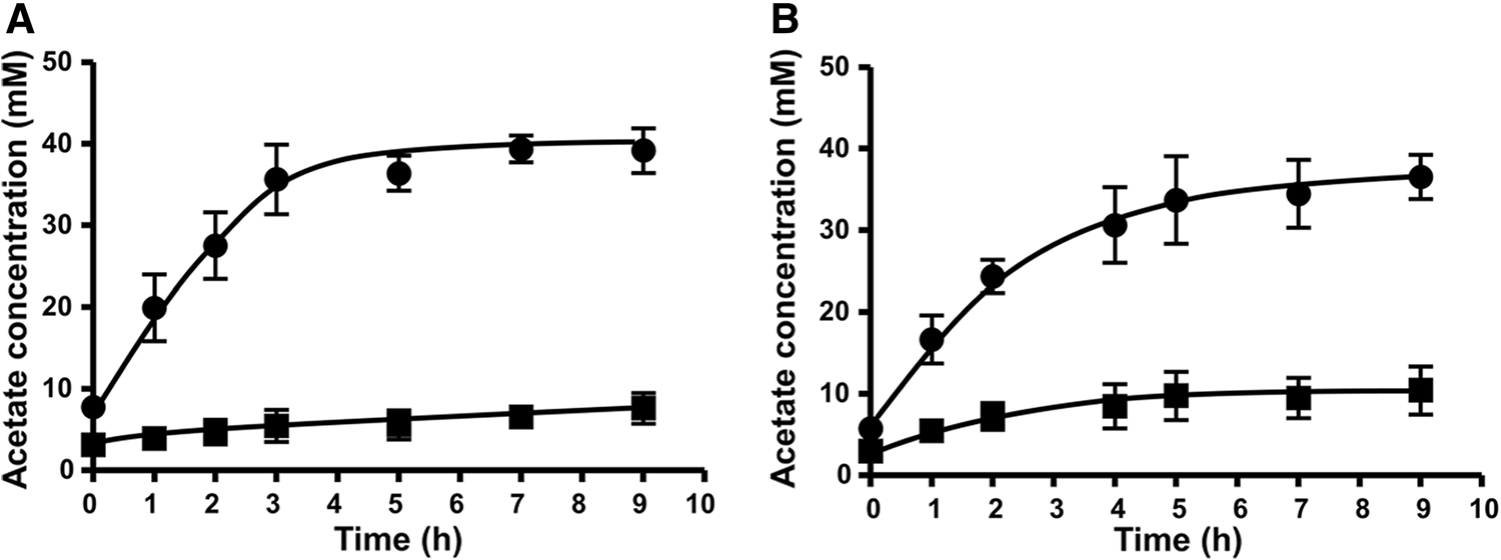
Conversion of H_2_ + CO_2_ to acetate by resting cells of *T. kivui* wild type (CO) and ∆*cooS*. The cells were grown on 25 mM glucose (**A**) or 25 mM glucose with 100% CO in the headspace (**B**) to the mid exponential phase. The harvested and washed cells were resuspended in imidazole buffer (50 mM imidazole, 20 mM MgSO_4_, 20 mM KCl, 20 mM NaCl, 4 mM DTE, 4 μM resazurin, pH 7) to a final protein concentration of 1 mg/ml with the addition of 50 mM KHCO_3_ in anoxic serum bottles. The resting cells were incubated with H_2_ + CO_2_ (80/20 [v/v], 1 × 10^5^ Pa) as a substrate in a shaking water bath at 66 °C. H_2_ + CO_2_ conversion to acetate was measured over the time. These experiments were performed in biological duplicates. Squares, *T. kivui* wild type (CO); circles, *T. kivui* ∆*cooS*

The aforementioned experiments clearly revealed that CooS is essential for growth on CO. However, CO is also an intermediate in the WLP, produced by the CODH/ACS from CO_2_. Therefore, it was of interest to study the effect of the deletion of *cooS* on acetate formation from H_2_ + CO_2_. To this end, cells were grown on either glucose alone or on glucose under a CO atmosphere (100%), harvested in the mid exponential growth phase and resting cells were prepared. As can be seen in Fig. 6, acetate was produced from H_2_ + CO_2_ and cells pre-grown in the presence of CO (plus glucose) had a slightly higher activity. Interestingly, acetate formation was drastically stimulated by *cooS* deletion in any case, demonstrating a role of CooS also in acetogenesis from H_2_ + CO_2_.

## Discussion

*T. kivui* is a thermophilic acetogen with a high potential as catalyst in carbon capture and storage as well as utilization (Müller 2019). It grows in synthetic media with high rates on H_2_ + CO_2_ and, although it had originally been described to not use CO as sole carbon and energy source, it was adapted to CO by subsequent transfer to media with increasing CO concentrations since it finally grew at 100% CO (Weghoff and Müller 2016). The molecular basis for this adaptation still remains elusive but here we have identified the CO dehydrogenase gene that is essential for growth on CO.

*T. kivui* has a gene encoding a monofunctional CO dehydrogenase, *cooS*, as well as a gene encoding the CO dehydrogenase subunit of the bifunctional CODH/ACS complex. After homologous production and purification from *T. kivui*, CooS was co-purified with CooF_1_, a protein that is suspected to mediate the electron transfer to Fd, the natural electron acceptor of CO dehydrogenases. AcsA forms a stable complex with AcsB, the acetyl-CoA synthase. Both complexes, CooSF_1_ and AcsAB, reduced MV but also Fd with CO as electron donor, albeit the AcsAB complex had much higher activities than the CooSF_1_ complex with both electron acceptors. AcsAB of *T. kivui* was more active than the purified and characterized bifunctional CODHs (AcsAB) of *A. woodii*, *Moorella thermoacetica* or *Clostridium formicoaceticum* with MV (27, 20 or 14 U/mg) or Fd as electron acceptor (112, 60, 14 U/mg) (Ragsdale et al. 1983). Acetogenic, monofunctional CODHs have not been purified and characterized yet. The activities of present monomeric CODHs were only measured with viologens as electron acceptor. If comparing the specific CO:MV oxidoreductase activity of CooSF_1_ from *T. kivui* to known monofunctional CODHs e. g. from *R. rubrum* or *D. vulgaris* (660 or 160 U/mg) (Ensign and Ludden 1991; Hadj-Saïd et al. 2015) the enzyme of *T. kivui* is less active. However, one should keep in mind that the assays were done with artificial electron acceptors or ferredoxin isolated from a mesophile. Although His-AcsAB of *T. kivui* was more active than His-CooSF_1_ of *T. kivui,* deletion of *cooS* clearly demonstrated that CooS is essential for growing on CO. The physiological importance of AcsAB in CO metabolism could not be addressed since all attempts to generate a CODH/ACS deletion mutant failed so far. Deletion of AcsAB will lead to a non-functional WLP. Some acetogens may grow on syngas in the absence of WLP, for example by producing hydrogen, but *T. kivui* requires the WLP also for the heterotrophic growth (Jain et al., 2020). Under autotrophic conditions, the WLP is essential. Thus, a knockout of *acsA* in *Clostridium autoethanogenum* led to a complete loss of autotrophy, i.e. growth on H_2_ + CO_2_ or CO (Liew et al. 2016a). *C. autoethanogenum* has two additional *cooS* genes. Deletion of *cooS*_*2*_ had no significant effect on autotrophic growth, whereas deletion of *cooS*_*1*_ led to a long lag phase, slower growth, lower OD and a shift in the product spectrum from acetate to ethanol. Thus, in this acetogen the monofunctional CODH’s are dispensable for autotrophic growth (Liew et al. 2016a). The same was observed for the monofunctional CODH’s in *Methanosarcina acetivorans* (Matschiavelli et al. 2012; Rother et al. 2007). In sharp contrast, deletion of *cooS* in *T. kivui* led to the complete loss of growth on CO, demonstrating that AcsA cannot compensate for a loss of CooS, despite of its higher CO oxidizing activity. This may have to do with regulatory effects. The physiological function of CODH/ACS is to reduce CO_2,_ not to oxidize CO, and this function may be downregulated in the cells.

Interestingly, and unexpectedly, acetate formation from H_2_ + CO_2_ was drastically increased in the *cooS* deletion strain. The precursor of acetate is acetyl-CoA which is also the central precursor for all the biosynthesis pathways in acetogens. Indeed, twice the amount of biomass was produced in a ∆*cooS* mutant of *C. autoethanogenum*. It was hypothesized that a deletion of *cooS* directs more CO_2_ to acetyl-CoA synthesis (Liew et al. 2016a). The same could be true for *T. kivui*, resulting in the observed enhanced acetate production. The molecular basis of this effect remains elusive but one scenario could be the following: during acetogenesis from CO_2,_ one CO_2_ is reduced to CO by CODH/ACS. Since CO is toxic to cells, it is kept caged in a tunnel in the enzyme before it is bound to a Ni-4Fe-4S center of the enzyme. But CO may also escape from the enzyme and the CooS then acts as a safety guard to detoxify cytosolic CO by oxidizing it to CO_2_, leading to a futile cycle. Deletion of *cooS* would then direct more CO to the direction of acetyl CoA.

## Supplementary Information

Below is the link to the electronic supplementary material.

Supplementary file1 (DOCX 1229 kb)

## Abbreviations

Fd: Ferredoxin
MV: Methyl viologen
CoA: Coenzyme A
WLP: Wood–Ljungdahl pathway
CODH/ACS: Carbon monoxide dehydrogenase/acetyl-CoA synthase
ATP: Adenosine triphosphate
4Fe-4S: Iron-sulfur cluster
SDS: Sodium dodecyl sulfate
Ni-4Fe-4S: Nickel–iron–sulfur center
5-FOA: 5-Fluoroorotate
PMSF: Phenylmethylsulfonyl fluoride
