# Supplementary material for: The monofunctional CO dehydrogenase CooS is essential for growth of Thermoanaerobacter kivui on carbon monoxide
Source: Extremophiles. 2021 Dec 17;26(1):4. doi: 10.1007/s00792-021-01251-y (PMC8683389; doi:10.1007/s00792-021-01251-y)
Supplement: Supplementary file 1 — Supplementary file1 (DOCX 1229 kb) [file 792_2021_1251_MOESM1_ESM.docx]

**Supplementary Information**

for the manuscript:

**The monofunctional CO dehydrogenase CooS is essential for growth of *Thermoanaerobacter kivui* on**

**carbon monoxide**

Surbhi Jain^1^, Alexander Katsyv^1^, Mirko Basen^2^ and Volker Müller^1#^

^1^Department of Molecular Microbiology & Bioenergetics, Institute of Molecular Biosciences, Johann Wolfgang Goethe University, Max-von-Laue-Str. 9, 60438 Frankfurt, Germany

^2^Microbiology, Institute of Biological Sciences, University of Rostock, 18059 Rostock, Germany

^#^correspondence address: Prof. Volker Müller, Department of Molecular Microbiology & Bioenergetics, Institute of Molecular Biosciences, Johann Wolfgang Goethe University, Frankfurt am Main, Germany; Phone: 49-6979829507; Fax: 49-69-79829306;

E-mail: [vmueller@bio.uni-frankfurt.de](mailto:vmueller@bio.uni-frankfurt.de)

**Fig. S1**

**Fig. S2**

**Fig. S3**

**Fig. S4**

**Fig. S5**


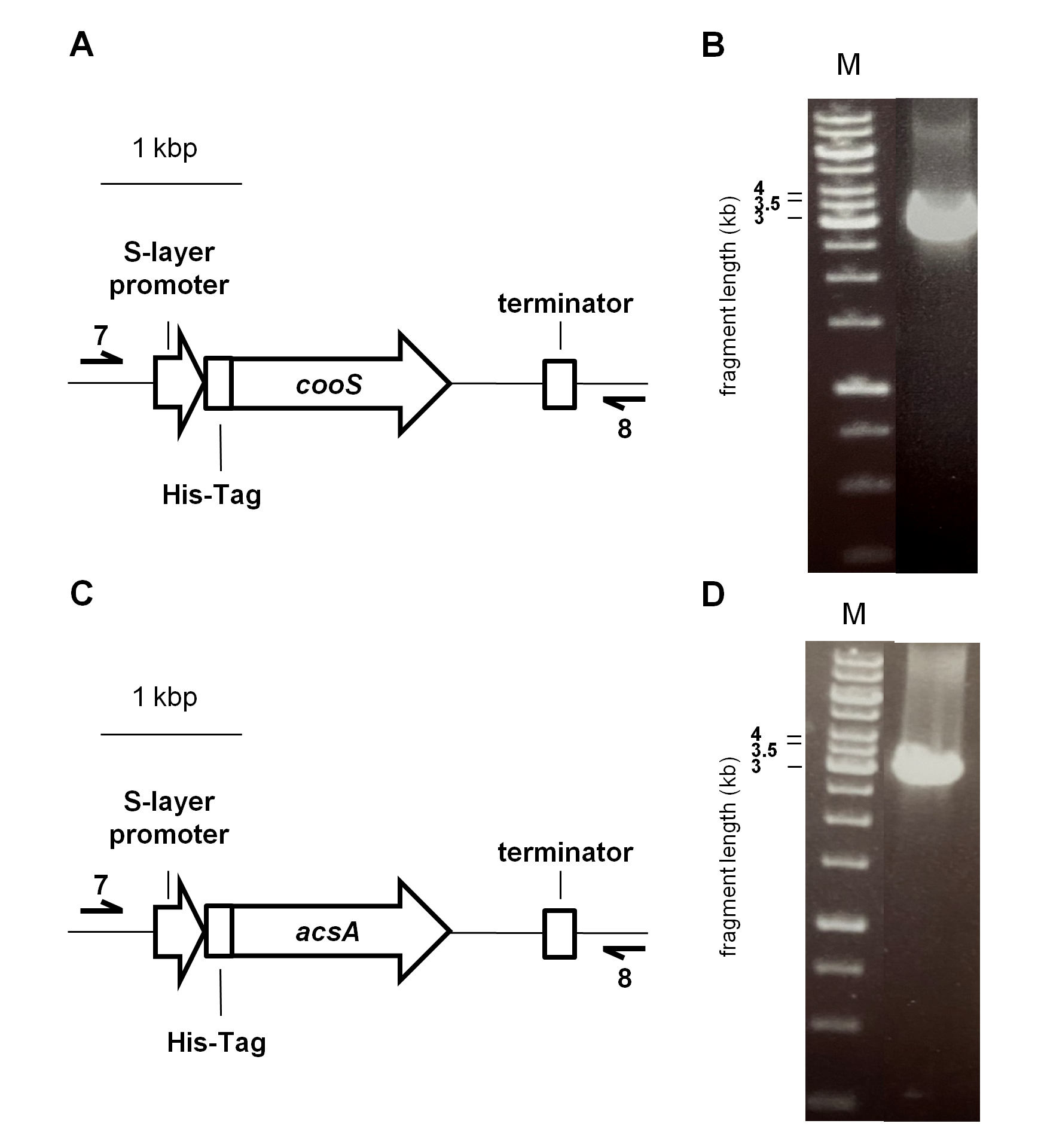


**Fig. S1 Verification of the *pMU131_His-cooS* and *pMU131_His-acsA* construct transformed in *T. kivui*.** To verify the nature of the plasmids *pMU131_His-cooS* or *pMU131_His-acsA* after propagation, *T. kivui* colonies were picked and the plasmid was checked by using primers that bind to the *pMU131* backbone and amplifying the complete *His-cooS* (A) or *His-acsA* (B). The resulting DNA fragments were 3110 bp (C) or 3083 bp (D). M, Gene Ruler 1 kb DNA ladder


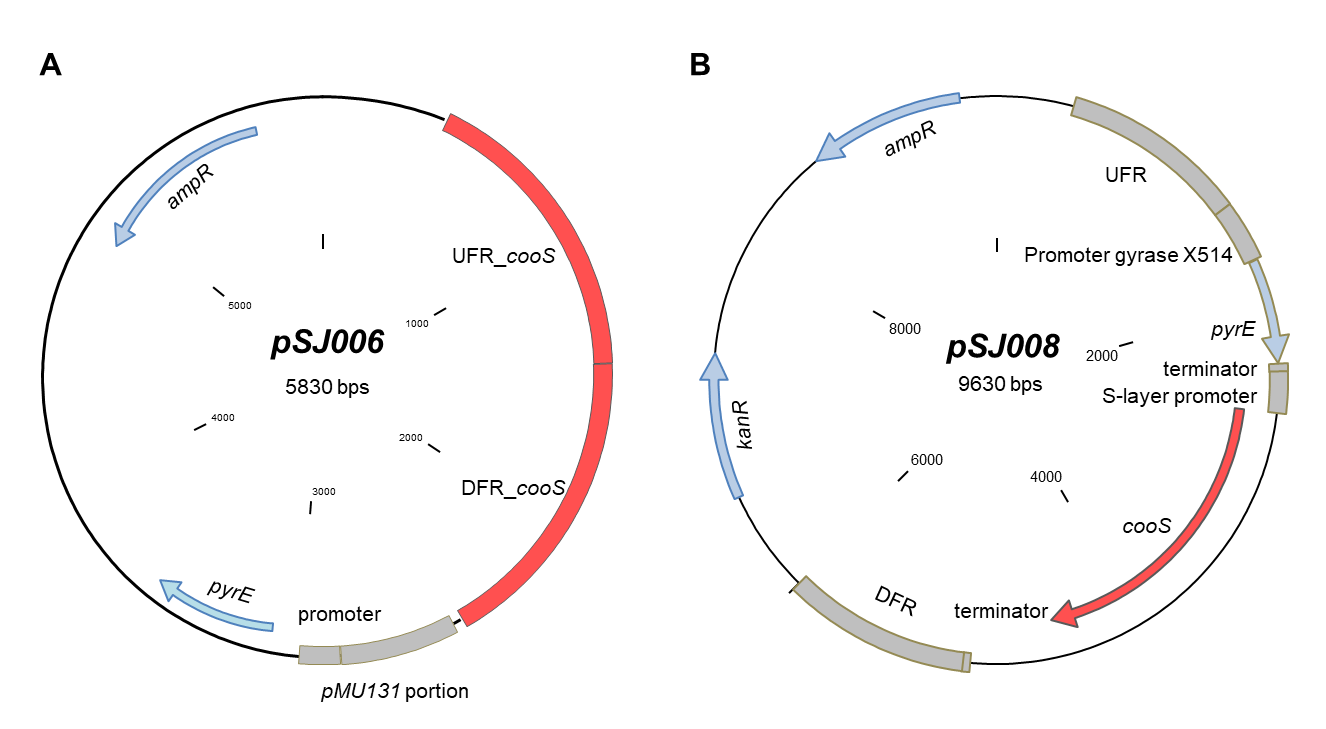


**Fig. S2 Physical map of plasmids *pSJ006* and *pSJ008***. The *pSJ006* (A) and *pSJ008* (B) construct was cloned as described in Materials & Methods. UFR, upstream flanking region; DFR, downstream flanking region


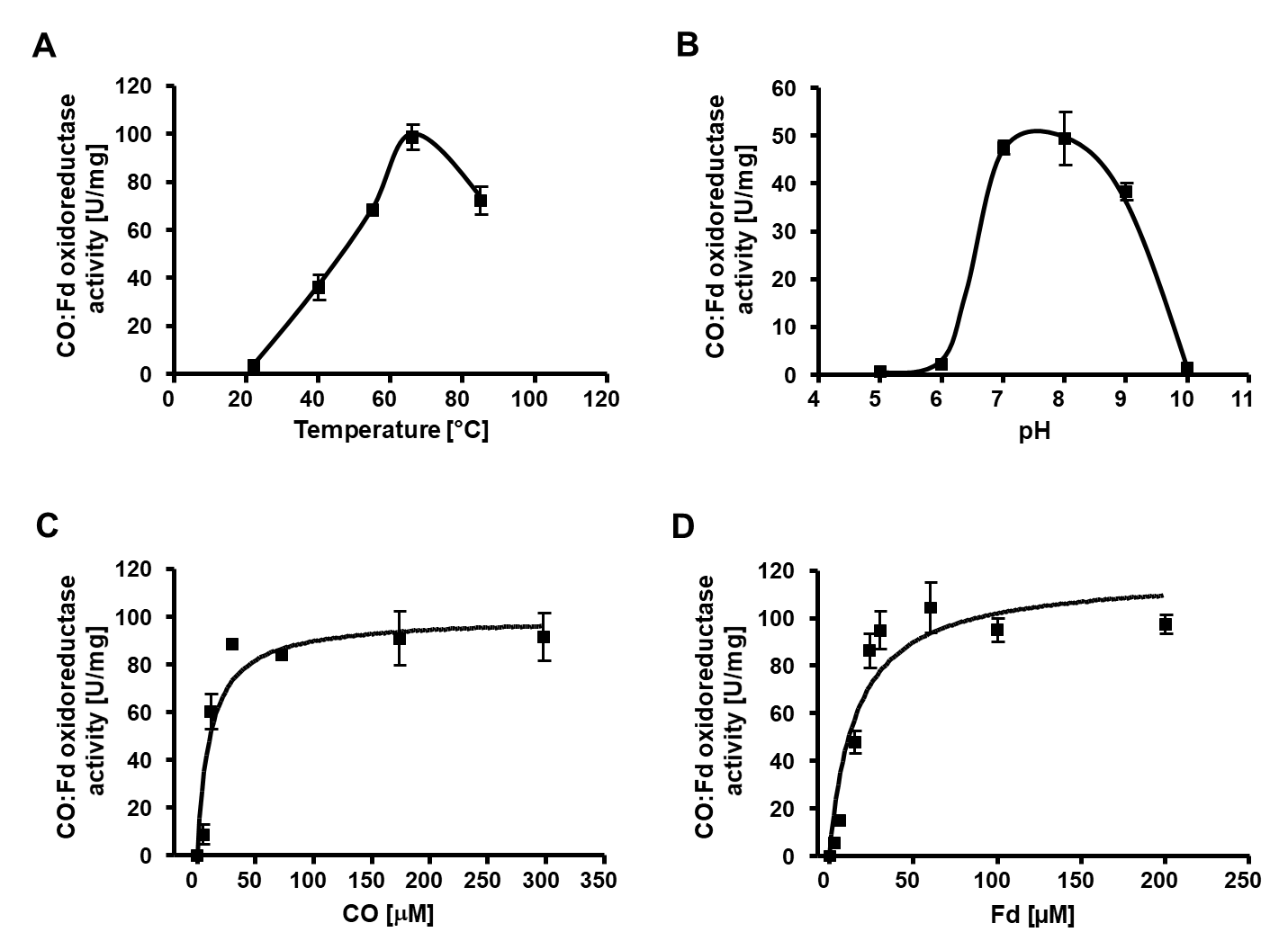


**Fig. S3 Temperature, pH, CO and Fd dependence of CO:Fd oxidoreductase activity as catalysed by His-AcsAB.** Temperature (A) or pH (B) effect of the CO-dependent Fd reduction was measured in 1.8-ml anaerobic cuvettes containing an overall liquid volume of 1 ml under a 100% CO atmosphere at 22 - 85 °C (A) or 66 °C (B). The assay contained 1 ml of buffer D (50 mM Tris/HCl, 10 mM NaCl, 2 mM DTE, 4 μM resazurin, pH 7.5) (A) or buffer F (50 mM Tris, 50 mM MES, 50 mM CHES, 50 mM CAPS, 50 mM Bis-Tris, 10 mM NaCl, 2 mM DTE, 4 µM Resazurin, pH 5 - 10) (B), 1 μg AcsAB and 30 μM Fd. CO (C) and Fd dependence (D) on AcsAB activity was measured in 1.8-ml anoxic cuvettes containing an overall liquid volume of 1 ml buffer D. The assay contained 1 μg AcsAB and different amounts of CO (C) or Fd (D). Shown is the average of two measurements from one representative experiment out of two independent replicates. Error bars represent the SEM.


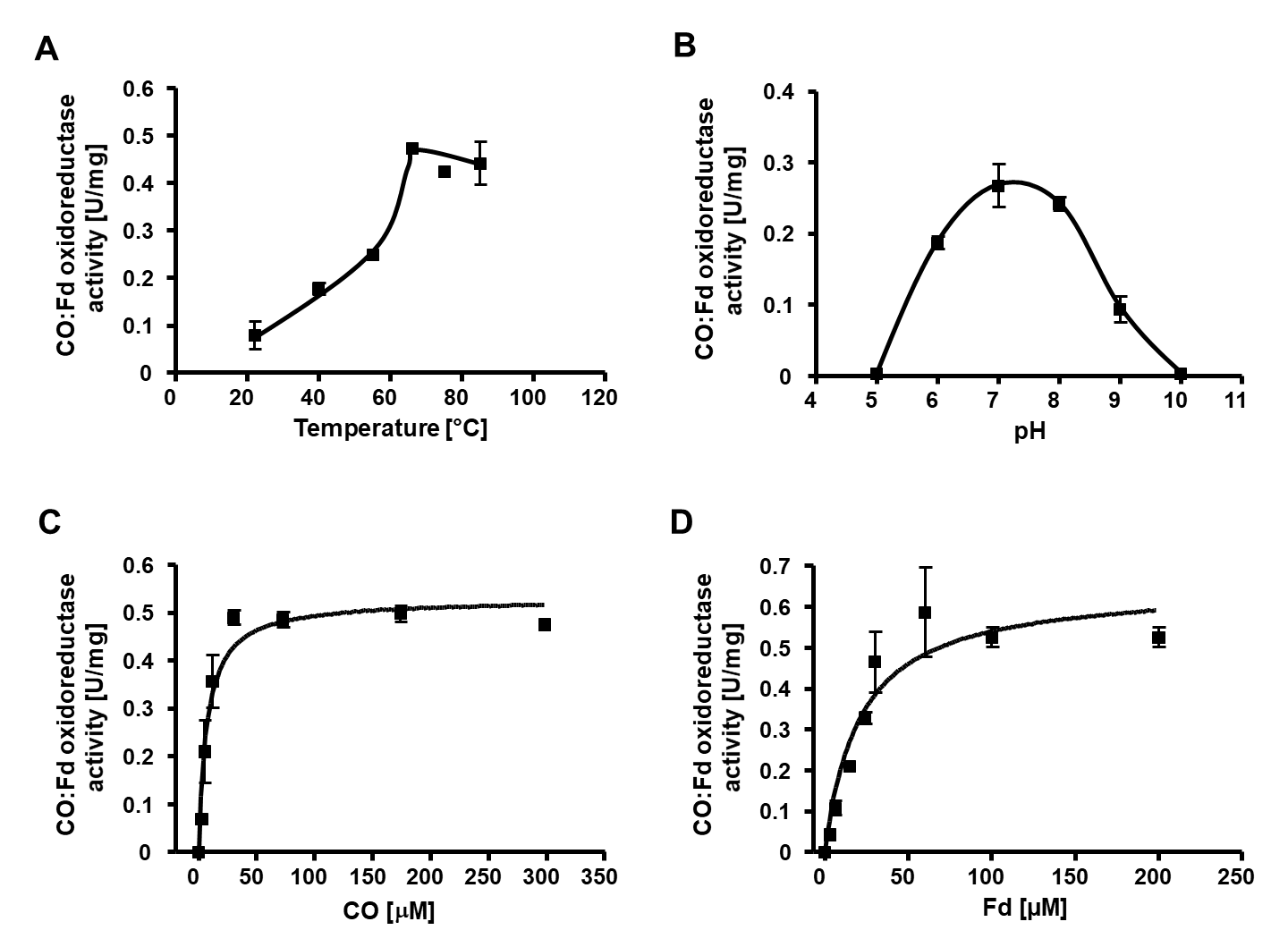


**Fig. S4 Temperature, pH, CO and Fd dependence of CO:Fd oxidoreductase activity as catalysed by His-CooSF_1_.** Temperature (A) or pH (B) effect on the CO-dependent Fd reduction was measured in 1.8-ml anaerobic cuvettes containing an overall liquid volume of 1 ml under a 100% CO atmosphere at 22 - 85 °C (A) or 66 °C (B). The assay contained 1 ml of buffer D (50 mM Tris/HCl, 10 mM NaCl, 2 mM DTE, 4 μM resazurin, pH 7) (A) or buffer F (50 mM Tris, 50 mM MES, 50 mM CHES, 50 mM CAPS, 50 mM Bis-Tris, 10 mM NaCl, 2 mM DTE, 4 µM Resazurin, pH 5 - 10) (B), 1 μg His-CooSF_1_ and 30 μM Fd. CO (C) and Fd dependence (D) on His-CooSF_1_ activity was measured in 1.8-ml anoxic cuvettes containing an overall liquid volume of 1 ml buffer D. The assay contained 1 μg His-CooSF_1_ and different amounts of CO (C) or Fd (D). Shown is the average of two measurements from one representative experiment out of two independent replicates. Error bars represent the SEM.


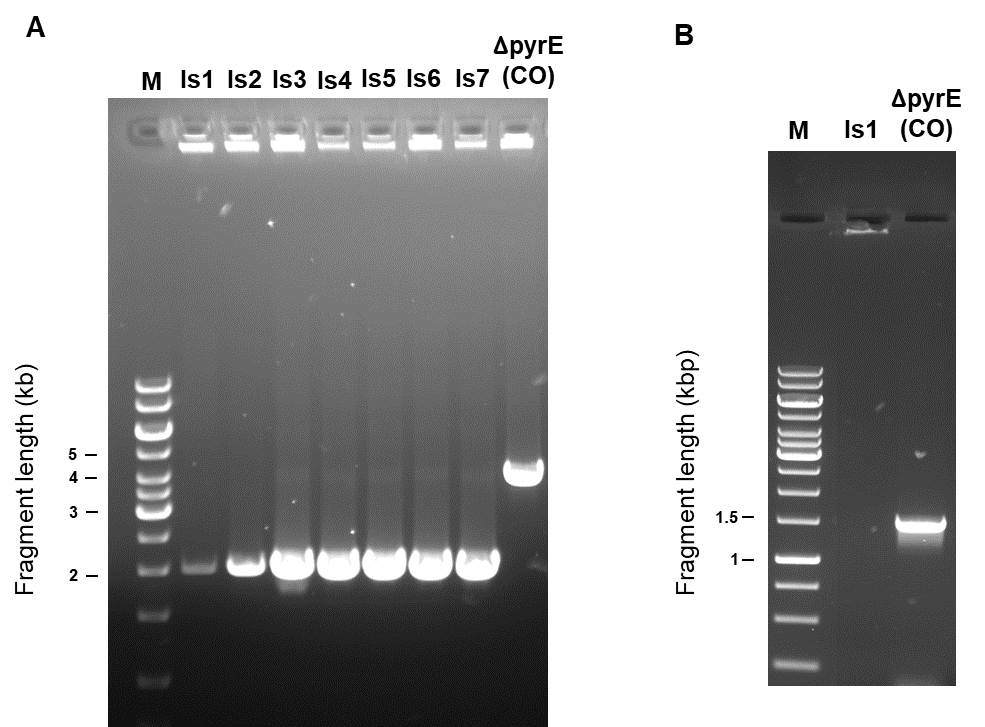


**Fig. S5 Verification of the *cooS* deletion in *T. kivui* ∆*pyrE via* PCR.** To verify the deletion of *cooS* after propagation, genomic DNA was isolated from *T. kivui* ∆*pyrE* (CO) or *T. kivui* ∆*cooS* isolates (isolates (Is) 1-7) and checked by PCR using primers which bind (A) outside the *cooS* gene (B) or inside the *cooS* gene. M, Gene Ruler 1 kb DNA ladder
